# Supplementary material for: Predicting visual function by interpreting a neuronal wiring diagram
Source: Nature. 2024 Oct 2;634(8032):113–23. doi: 10.1038/s41586-024-07953-5 (PMC11446822; doi:10.1038/s41586-024-07953-5)
Supplement: Supplementary file 6 — CRF and ERF predictions for individual TmY4 and TmY9 cells. Analogous to Supplementary Data 3, but for TmY target types. Shown are the top four monosynaptic pathways, the strongest pathway passing through each of the top ten intermediary types (ranking from Extended Data Fig. 7), and the trisynaptic pathway Tm1–TmY–Dm3–TmY (see the section entitled Prediction of spatial normalization). [file 41586_2024_7953_MOESM6_ESM.zip › DataS4/TmY9qΓèÑ/720575940661166977.pdf]

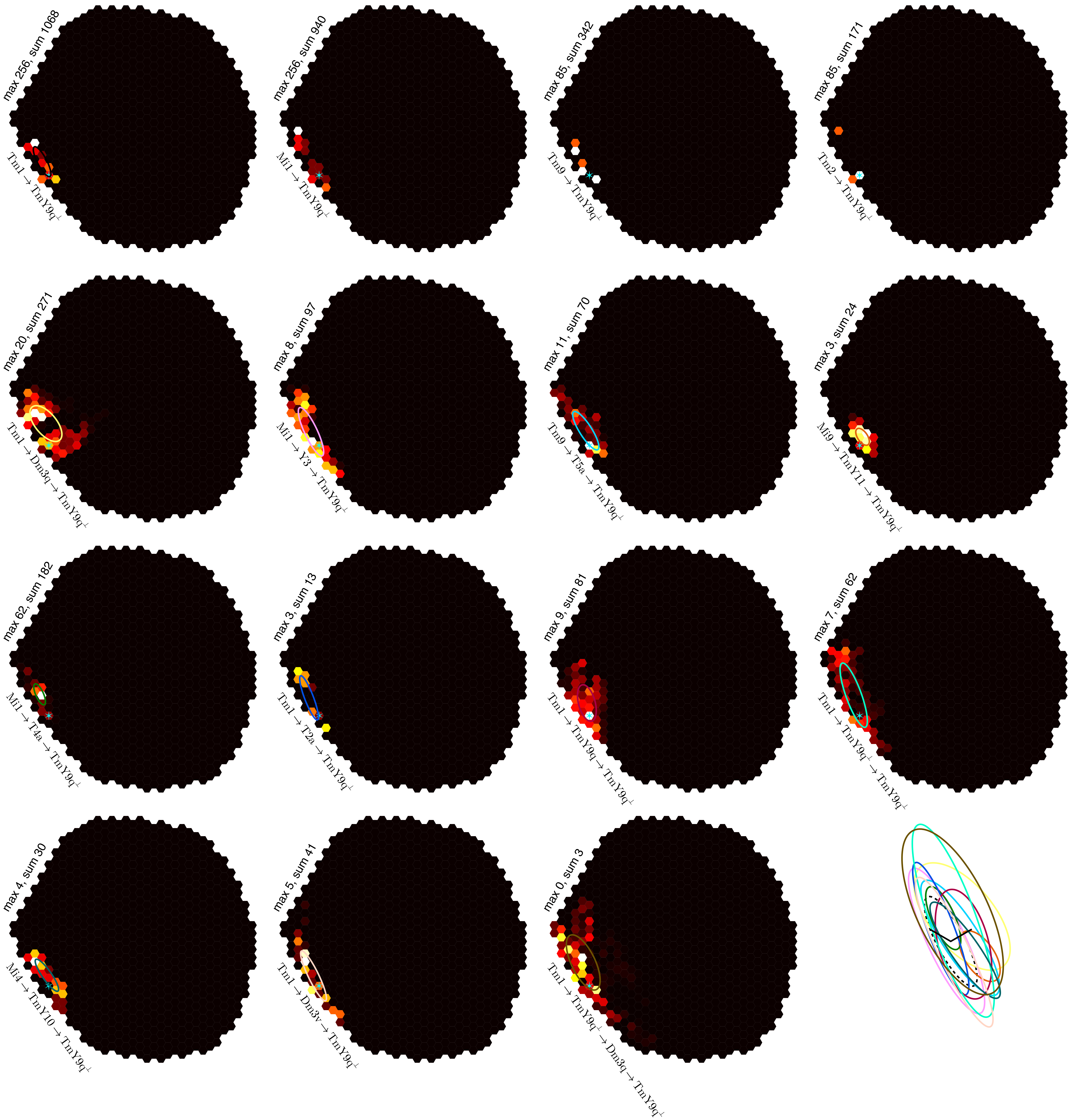
max 256, sum 1068
Tm1 → TmY9q⊥
max 256, sum 940
Mi1 → TmY9q⊥
max 85, sum 342
Tm9 → TmY9q⊥
max 85, sum 171
Tm2 → TmY9q⊥
max 20, sum 271
Tm1 → Dm3q → TmY9q⊥
max 8, sum 97
Mi1 → Y3 → TmY9q⊥
max 11, sum 70
Tm9 → T5a → TmY9q⊥
max 3, sum 24
Mi9 → TmY11 → TmY9q⊥
max 62, sum 182
Mi1 → T4a → TmY9q⊥
max 3, sum 13
Tm1 → T2a → TmY9q⊥
max 9, sum 81
Tm1 → TmY9q → TmY9q⊥
max 7, sum 62
Tm1 → TmY9q⊥ → TmY9q⊥
max 4, sum 30
Mi4 → TmY10 → TmY9q⊥
max 5, sum 41
Tm1 → Dm3v → TmY9q⊥
max 0, sum 3
Tm1 → TmY9q⊥ → Dm3q → TmY9q⊥
